# Genomic signatures of local adaptation reveal source-sink dynamics in a high gene flow fish species

**DOI:** 10.1038/s41598-017-09224-y

**Published:** 2017-08-17

**Authors:** Katherine Cure, Luke Thomas, Jean-Paul A. Hobbs, David V. Fairclough, W. Jason Kennington

**Affiliations:** 1 UWA Oceans Institute & School of Plant Biology, The University of Western Australia, Crawley, 6009 WA Australia; 2 Australian Institute of Marine Science, Crawley, 6009 WA Australia; 3 Hopkins Marine Station, Stanford University, California, 93950 USA; 4 Department of Environment and Agriculture, Curtin University, Bentley, 6102 WA Australia; 5 Western Australian Fisheries and Marine Research Laboratories, Department of Primary Industries and Regional Development, Government of Western Australia, P.O. Box 20, North Beach, 6920 WA Australia; 6 Centre for Evolutionary Biology, School of Animal Biology, The University of Western Australia, Crawley, 6009 WA Australia

## Abstract

Understanding source-sink dynamics is important for conservation management, particularly when climatic events alter species’ distributions. Following a 2011 ‘marine heatwave’ in Western Australia, we observed high recruitment of the endemic fisheries target species *Choerodon rubescens*, towards the cooler (southern) end of its distribution. Here, we use a genome wide set of 14 559 single-nucleotide polymorphisms (SNPs) to identify the likely source population for this recruitment event. Most loci (76%) showed low genetic divergence across the species’ range, indicating high levels of gene flow and confirming previous findings using neutral microsatellite markers. However, a small proportion of loci showed strong patterns of differentiation and exhibited patterns of population structure consistent with local adaptation. Clustering analyses based on these outlier loci indicated that recruits at the southern end of *C*. *rubescens*’ range originated 400 km to the north, at the centre of the species’ range, where average temperatures are up to 3 °C warmer. Survival of these recruits may be low because they carry alleles adapted to an environment different to the one they now reside in, but their survival is key to establishing locally adapted populations at and beyond the range edge as water temperatures increase with climate change.

## Introduction

The majority of marine species exist as discrete adult populations that are connected by a dispersive larval stage^1^. The need to identify source-sink dynamics of fish populations for fisheries and conservation management^2, 3^ has gained particular importance in light of recent species distribution shifts in response to climate change^4–6^. Distributional shifts can act as a buffer against potential extinctions from rising temperatures^7^ by allowing access to cooler areas and opening novel habitats for expansion^8, 9^. But such shifts require that populations can disperse and successfully adapt to the novel local environmental conditions^10–12^. Understanding larval connectivity patterns and the local adaptive capacity of populations is therefore crucial for assessing species potential for distributional shifts in response to climate change.

A recent ‘marine heatwave’ along the coast of Western Australia (WA) drastically changed local oceanographic conditions, simulating future climate change scenarios^13^. Oceanography in WA is largely governed by the poleward flowing Leeuwin Current (LC), which during this heatwave was stronger than in the two previous centuries, increasing maximum sea-surface temperatures by up to 3 °C along the entire WA coast^13, 14^. This anomalous oceanographic event resulted in mortality to fishes, crustaceans and corals^15, 16^, and numerous ecological changes^17^. High temperatures and strong LC flow were largely maintained during 2012 and 2013 when an unusually high recruitment event of the baldchin groper *Choerodon rubescens*, a highly targeted fish species endemic to WA, was documented towards its cooler (southern) range edge^18^. This recruitment event was viewed as a possible indication of a poleward distributional shift in response to warming oceans and stronger LC flow, but such high recruitment events are often episodic and may fail to establish the new populations necessary for such a shift to occur^19^. Identifying the origin of these recruits and assessing whether there is evidence for local adaptive capacity in this species can assist our understanding of source-sink dynamics and the potential response of this commercially important species to rapid environmental change. This information has important implications for the design of conservation management measures in this species and the large number of species undergoing distributional shifts at a global scale^4, 6, 20^.

A previous study using neutral microsatellite markers indicated that populations of *C*. *rubescens* are characterized by high levels of gene flow^21^ maintained primarily by a highly dispersive larval phase and counter-flowing currents^13, 22^. High levels of gene flow and associated lack of population structure make it difficult to assign recruits to their natal populations^23^, particularly given the challenges in obtaining complete sampling of the putative parent populations that is necessary for parentage studies^24^. However, population genomic studies involving high numbers of single-nucleotide polymorphisms (SNPs) have revealed that despite considerable gene flow and an apparent lack of population structure, many fish species show significant levels of differentiation at highly structured ‘outlier’ loci that are putatively under directional selection^25–28^. This is particularly the case when samples are collected across strong environmental gradients^29^. Genome-wide scans for outlier loci putatively under selection can therefore be useful for assigning recruits in high gene flow species where neutral genetic markers fail to show population structure^30^.

In this study, we specifically test for the presence of outlier loci in adult populations of *C*. *rubescens*, a high-gene flow endemic species distributed across a temperate-tropical transition zone that could be used to assign recruits to their most likely natal population. This study presents two sets of information important for developing climate adaptive management measures for this endemic fisheries target species: (1) a scan for genomic signatures of local adaptation, and (2) identification of the geographical origin of recruits towards the cooler range-edge of this species, during an anomalous marine heatwave event. Since shifts in distributions of marine species occur mostly along ecosystems influenced by poleward boundary currents^9, 17, 20^, the applicability of the results from this study can be extended to global conservation management and bring insight into the exchange of larvae between populations that differ in their locally adaptive genetic signatures.

## Results

### Marker genotyping and detection of outlier loci

**Figure 1:**
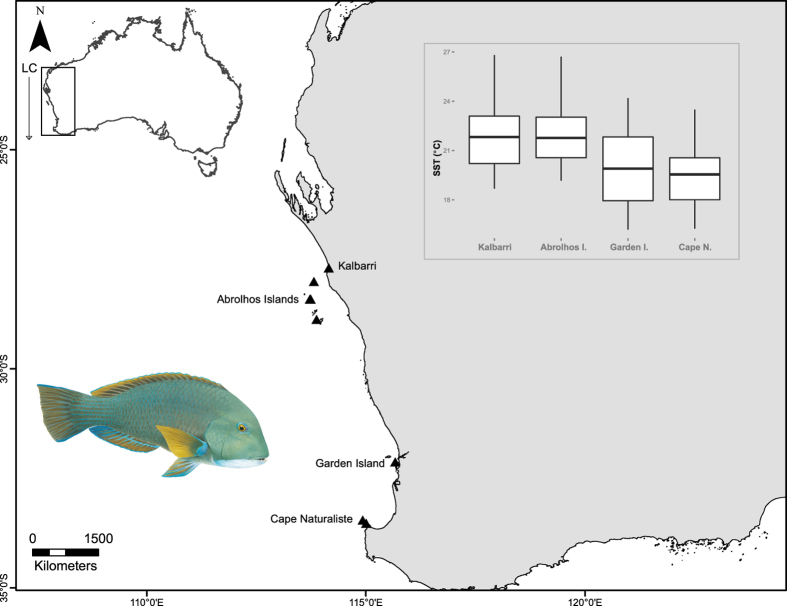
Map of Western Australia (WA) showing locations sampled across the geographical range of *Choerodon rubescens* (triangles represent reefs sampled within locations). Geographical range is represented by a square inset over the map of Australia in the top left corner. Boxplot inset shows long-term monthly average sea surface temperatures (SST °C) at each location; data from MODIS-AQUA satellite (Goddard Earth Sciences Data and Information Services Center GES DISC, NASA); solid black lines represent median SST (2002–2013) and box boundaries upper and lower quartiles. LC: Leeuwin Current flow along the WA coast. *C*. *rubescens* illustration © R. Swainston/anima.net.

Single-nucleotide polymorphism (SNP) loci were called across 65 *C*. *rubescens* samples collected from four different locations spanning the species’ geographical range (Fig. 1; Table 1). A total of 50 279 SNPs were produced of which 14 559 were retained following the implementation of selection criteria (see methods). Of these loci, 1.9% were identified as outliers putatively under directional selection (n = 282) across 10 independent runs of outlier analyses using LOSITAN^31^ and assuming a false discovery rate (FDR) of 0.1^31^. Removal of these outliers and those under balancing selection (3 222 loci, 22.13% of all loci), resulted in a neutral dataset consisting of 11 055 loci. None of the loci in the neutral data set deviated significantly (*p* < 0.05) from Hardy Weinberg equilibrium consistently in any of the sampling locations. Levels of linkage disequilibrium (LD) were low overall with less than 1% of neutral loci showing significant LD (*p* < 0.05), after correcting for multiple comparisons via the Benjamini & Hochberg method^32^. For these reasons, all 11 055 loci in the neutral data set were retained for further analyses.

**Table 1:** Collection and sample details including study locations with geographic coordinates, average sea-surface temperature during winter (wSST °C) and summer (sSST °C) (2002–13), total number of fish collected (N), mean fish size (mm total length, L_T_) and collection date.

Location	Latitude (°S)	Longitude (°E)	wSST	sSST	N	Mean size (mm L_T_)	Collection date
Kalbarri	27.6937	114.1500	20.83 °C	23.14 °C	11	469.09	Mar 2013
Abrolhos Islands	28.8688	113.8730	21.39 °C	22.67 °C	29	462.21	Oct 2012-Apr 2013
Garden Island	32.1184	115.6627	17.98 °C	22.17 °C	14	111.07	Jan 2013
Cape Naturaliste	33.5193	115.0004	18.62 °C	20.40 °C	11	524.82	Jan-Feb 2013

Temperature data from MODIS-AQUA satellite (Goddard Earth Sciences Data and Information Services Center GES DISC, NASA).

Genome-wide scans for selection with both BAYESCAN^33^ and BAYESCENV^34^ identified a lower number of outlier loci than LOSITAN, but these loci were always a subset of those identified via LOSITAN, demonstrating high consistency between methods. BAYESCAN^33^ identified 0.06% of all SNP loci (n = 9) as being under directional selection. Outlier analyses with BAYESCENV^34^ identified 0.15% of all SNP loci (n = 22), and further revealed a significant association with average sea surface temperatures (SST) along the latitudinal gradient sampled during both summer (mean *q*-*value* 0.0174, n = 11) and winter (mean *q*-*value* 0.0165, n = 12).

### Population genetic structure and recruit assignment

**Figure 2:**
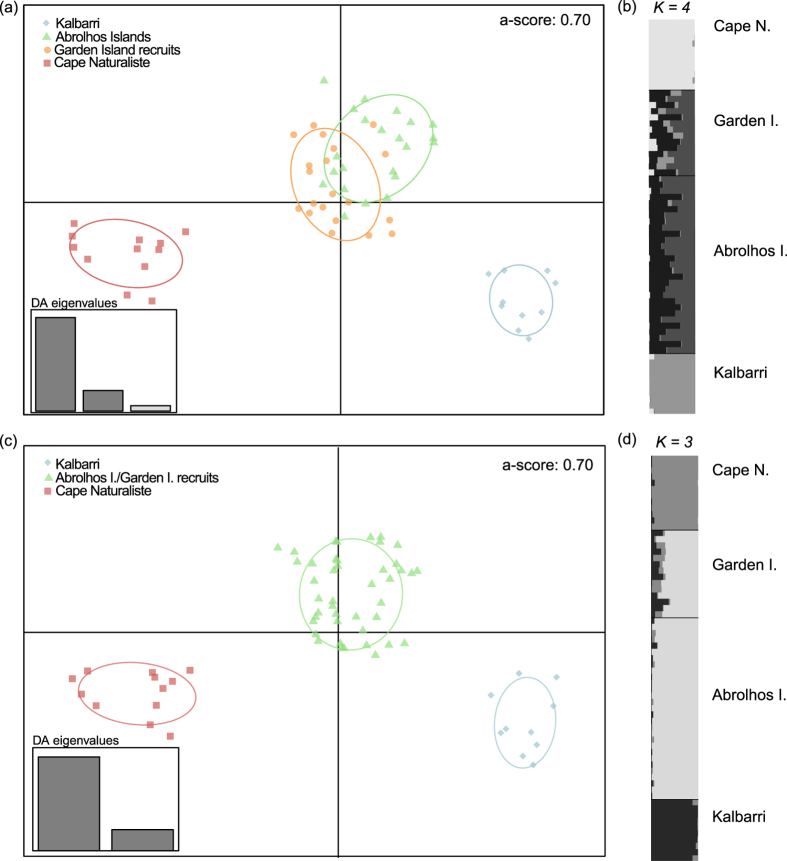
Structure of outlier loci (n = 282) from *Choerodon rubescens* populations. Two discriminant analysis of principle components (DAPC) are presented: (**a**) shows original populations as clusters (*K* = 4), and (**b**) the scenario with *K* = 3 selected as the optimal number of clusters via Bayesian analyses (BIC, see Supplementary Fig. S3 for *K* selection information). For each DAPC the associated alpha score (a-score) is shown; geographical origin of each population is depicted by colours and 95% inertia ellipses; individual genotypes are represented by different shaped dots (diamonds, triangles, circles and squares); eigenvalues show the amount of genetic information contained in each successive principal component. (**b**) and (**d**) show bar plots from Bayesian clustering analyses using STRUCTURE for *K* = 4 and *K* = 3 respectively; each individual is represented by a vertical column partitioned into *K* segments (grayscale); see Appendix 4 for plots of log probability L(*K*) and *ΔK* across different values of *K*. Fish at Kalbarri, Abrolhos, and Cape Naturaliste are adults (≥350 mm L_T_), while the population at Garden Island is represented exclusively by recruits (≤130 mm L_T_, 0 + yrs). See Table 1 for sample details.

As expected, we found substantial differences in population genetic structure patterns inferred from analyses based on the neutral *vs* outlier loci datasets (Fig. 2; Supplementary Fig. S2). Discriminant analysis of principle components (DAPC)^35, 36^ using the neutral dataset showed no evidence of population structure, suggesting *C*. *rubescens* is composed of a single panmictic population (see Supplementary Fig. S2). Bayesian analyses (BIC) revealed *K* = 1 as the most likely number of genetic clusters (see Supplementary Fig. S3), and were confirmed via results from STRUCTURE HARVESTER^37^, which showed that log-likelihood values were highest for one group *K* = 1 and declined exponentially as the number of groups increased (see Supplementary Fig. S4). The absence of population structure is further confirmed by the low *F*_ST_ values found across all neutral loci (*F*_ST_ = 0.0047).

In contrast, analyses performed on outlier loci provided evidence of significant population structure (Fig. 2), which could then be used for recruit assignment^38^. DAPC plots with *a priori* information of *K* = 4 (the number of populations sampled), revealed distinct populations for Cape Naturaliste and Kalbarri, which separated from a group of overlapping populations formed by Abrolhos Islands adults and Garden Island recruits (Fig. 2a). Results from STRUCTURE^39^ for *K* = 4 showed the same patterns of population structure to those suggested by the DAPC plot (Fig. 2b). Bayesian information criterion (BIC) methods revealed *K* = 3 as the optimal number of clusters in the outlier dataset (see Supplementary Fig. S3). When DAPC plots were produced with *a priori* information of *K* = 3 from the BIC method, all fourteen Garden Island recruits were assigned to the adult population at the Abrolhos Islands (Fig. 2c). Results obtained from STRUCTURE HARVESTER^37^ supported *K* = 3 as the most likely number of clusters among populations, and also indicated that the Abrolhos Islands adults and Garden Island recruits were genetically homogeneous (Fig. 2d). Further support for the homogeneity of genetic structure between these two populations was provided by STRUCTURE HARVESTER results on a subsample of the data including only Abrolhos Islands adults and Garden Island recruits, which identified *K* = 1 as the most likely number of genetic clusters (see Supplementary Fig. S4).

### Gene function of outlier loci

Results from our NCBI Blast search linked 5% of the outlier loci (13 outlier loci identified by LOSITAN and one outlier locus identified via the three outlier identification methods used -LOSITAN, BAYESCAN, and BAYESCENV) to proteins involved in important cellular processes such as growth and metabolism, membrane transport and signal transmission (Table 2). Other outlier loci did not meet our specified selection criteria for significant alignment with publicly available sequences (see methods section).

**Table 2:** List of outlier loci selected from BLASTn results together with alignment information: locus name, genetic differentiation (*F*_ST_), BLASTn top hit, sequence coverage (%) and e-value.

Locus	*F*_ST_	BLASTn Top Hit	Coverage (%)	E-value	Function	Reference
TP581	0.284	*Thamnophis sirtalis*: acetylserotonin O-methyltransferase-like (ASMTL)	98	2.14 * 10^−18^	Synthesis of melatonin	86
**TP2626**	0.423	*Labrus bergylta*: seizure threshold 2 homolog (mouse) (szt2)	100	5.96 * 10^−19^	Nervous system	87
TP10437	0.123	*Larimichthys crocea*: putative sodium-coupled neutral amino acid transporter 8	100	1.66 * 10^−19^	Membrane transport & signals	88
TP16126	0.178	*Takifugu rubripes*: erythrocyte band 7 integral membrane protein-like	100	5.96 * 10^−19^	Membrane transport & signals	89
TP33552	0.070	*Austrofundulus limnaeus*: Rho guanine nucleotide exchange factor (GEF) 37 (arhgef37)	80.5	2.81 * 10^−12^	Membrane transport & signals	90
TP42559	0.123	*Stegastes partitus*: phosphatidylinositol-4-phosphate 5-kinase, type I, beta (pip5k1b)	95	2.77 * 10^−17^	Growth & metabolism	47
TP55758	0.105	*Monopterus albus*: HECT domain E3 ubiquitin protein ligase 1 (hectd1)	80.5	2.81 * 10^−12^	Membrane transport & signals	91
TP73052	0.070	*Stegastes partitus*: early endosome antigen 1 (eea1)	100	1.29 * 10^−15^	Waste storage regulation	92
TP86444	0.132	*Labrus bergylta*: protein kinase C-binding protein NELL1-like	100	1.28 * 10^−20^	Growth & metabolism	46
TP98903	0.188	*Lates calcarifer*: uncharacterized LOC108874954)	100	2.77 * 10^−17^	—	
TP109927	0.204	*Labrus bergylta*: myelin transcription factor 1 like (myt1l)	96	7.76 * 10^−13^	Nervous system	93
TP118519	0.182	*Labrus bergylta*: multiple EGF like domains 10 (megf10)	100	1.28 * 10^−20^	Muscle growth	94
TP133930	0.132	*Fundulus heteroclitus*: collagen and calcium-binding EGF domain-containing protein 1-like	85	2.79 * 10^−12^	Embryogenesis	95
TP137584	0.070	*Lates calcarifer*: metastasis suppressor protein 1-like	78	1.67 * 10^−14^	Growth & metabolism	45

Also shown is the physiological function associated with each top hit and a reference. Only top hits with ≥50% coverage and ≤e^−10^ were selected during BLASTn. Bold typeface indicates outlier loci that were identified via LOSITAN, BAYESCAN and BAYESCENV methods.

## Discussion

Population genetic analyses using a dataset comprised of 11 055 neutral single nucleotide polymorphisms identified an absence of genetic structure in populations of the highly targeted fish species *C*. *rubescens* across 12 degrees of latitude in Western Australia. By contrast, genome-wide scans for selection identified 282 outlier loci putatively under directional selection that showed significant population structure between adult sampling regions and thus could be used to assign juveniles from an anomalous recruitment event at the southern edge of the species distribution to a population over 400 km to the north at the Abrolhos Islands, where average sea surface temperatures are up to 3 °C warmer. It is important to note, however, that while our results indicate that the Abrolhos Islands are part of the same genetic source population as the Garden Island recruits, sampling of the adult populations was too coarse to definitely pinpoint the geographic origin of these recruits. Increased strength of poleward current flow, together with warmer temperatures associated with a recent marine heatwave, may have assisted larvae originating from warm water sites to disperse to, settle and persist at the cooler end of the species distribution. Recruit settlement was traced to autumn and winter 2012 (see Supplementary Fig. S1) when the LC is strongest^40^, and sea-surface temperatures were above long-term averages (see Supplementary Fig. S5). Although this seems to be the most likely scenario, an alternative scenario may be that *C*. *rubescens* recruits at the cooler (southern) end of the species range may have originated from a highly mixed larval pool derived from populations spread throughout the species range and that intense post-settlement selection in response to warmer than long-term average water temperatures at the site where recruits were collected may have driven the observed genetic similarity in outlier loci between the recruits at Garden Island and adult fish from warmer water sites to the north.

We found contrasting patterns of genetic variation in neutral and outlier loci suggesting the presence of possible local adaptation amidst high levels of gene flow in an important endemic fishery species in WA^41^. Lack of structure in neutral loci indicates that *C*. *rubescens* populations are connected via high levels of gene flow across the thermal and habitat gradients sampled, and between offshore islands and the mainland, consistent with a previous microsatellite study of this species^21^. Larval connectivity is promoted by a pelagic larval phase and counter-flowing current systems along south-western WA which promote larval mixing (poleward flowing Leeuwin Current (LC) *vs* the northward flowing inshore currents of Ningaloo and the Capes^13, 22^). Despite these high levels of gene flow, outlier loci revealed patterns of population structure that support post-settlement selection and suggest that strong selective forces are acting on a mixed pool of recruits resulting in the selective mortality of juveniles originating from cooler environments. Although strong selection appears to be acting at a local scale, connectivity is present between cooler and warmer water populations promoted by counter-flowing currents in WA, making it difficult to distinguish between the processes of dispersal and selection. While the selective forces causing local adaptation are unknown, an environmental gradient in sea surface temperature is a likely factor^42, 43^. The association of outlier loci with genes that play a direct role in growth and metabolism^44–47^, processes that are highly regulated by temperature in marine teleosts^48, 49^, provides some support for this hypothesis. It is further supported by the significant association between outlier loci and sea surface temperature found in this study (*q*-*values* < 0.05). Several outlier loci also had best blast hits with genes associated with temperature. For example, genes of the ubiquitin family and neural epidermal growth factor-like like (NELL) have been shown to upregulate in response to heat stress^50–52^. However, we cannot rule out the influence of other factors correlated with temperature such as latitude, depth and habitat^53, 54^.

Using outlier loci to assign recruits to source populations provides a novel and powerful approach to understanding population connectivity and the evolution of geographic ranges in marine species with a dispersive larval stage. The genetic mismatch at outlier loci between recruits at Garden Island and adults from a neighbouring population at Cape Naturaliste with a similar temperature profile, suggests the possibility that recruits may be maladapted to their new environment at the southern range edge^55, 56^, once temperatures return to normal conditions following the heatwave. Recruit ageing demonstrated that juveniles have survived one winter of warmer waters (see Supplementary Fig. S1 and Table 1) suggesting they will persist at least as long as the water temperatures remain sufficiently warm. However, to clarify the fate of these recruits and determine whether they are adapted to the local environment, requires tracking this cohort through time, determining their survival across subsequent colder winters and growth into reproductive adults, and monitoring for potential changes in the genetic composition of the population. If there is high local adaptation across the environmental gradient of *C*. *rubescens* range, the introduction of genotypes adapted to warmer waters could result in allele swamping^57^ and reduce local fitness at the southern range edge, with negative demographic consequences. On the other hand, because local environments are changing as a result of climate change, the introduction of genes successful in lower latitude populations (via either dispersal or selection) could actually increase the adaptive capacity of populations at higher latitudes^58^. As *C*. *rubescens* has relatively long generation times (long life span, and at least 7 years for sex change to mature males^59, 60^), evolutionary responses to increasing temperatures are likely to be slow and may require frequent introductions of adaptive variation in order to maintain local fitness optima^61^. Expansion of our sampling to include the full range of *C*. *rubescens* with a focus on specifically determining local adaptive capacity in response to temperature is recommended.

We found evidence of adaptation to local environmental gradients amidst high levels of gene flow and poleward larval connectivity in a highly targeted endemic species in WA. These characteristics suggest an adaptive capacity to changing environments via successful colonization of novel habitats at cooler locations^10, 62^ along the Capes region, which are becoming increasingly warmer with climate change^63^. Poleward range-shift facilitation via successful colonization and adaptation may therefore be likely for this and other fish species in the region. However, because the LC is expected to weaken in the next five decades^64^, the Capes counter-current will likely flow at greater strength promoting higher self-recruitment and/or northward larval dispersal^65^, and could ultimately restrict the extension of species to their cooler range margins in this region (*i*.*e*. the Capes region). Coupled with increasing temperatures at the warmer (northern) range edge, geographical distributions could ultimately be reduced rather than extended. It is therefore crucial to better understand dispersal pathways across the full range of *C*. *rubescens*, with emphasis on the Abrolhos Islands, where it is currently most abundant and which appears to be an important larval source. This study applied powerful genomic techniques to better understand processes of dispersal and recruitment in a high gene flow species and highlights the importance of such studies in monitoring changes in source-sink dynamics associated with a rapidly changing climate.

## Methods

### Study species

*Choerodon rubescens* (Labridae) is a large bodied wrasse and an important fisheries target^41^, distributed along a latitudinal gradient spanning ~1 400 km of the WA coast in habitats that range from tropical coral to temperate rocky-kelp reefs^66^ (Fig. 1).The relative abundance of *C*. *rubescens* is highest towards the centre of its geographical range at the Abrolhos Islands, and low towards both northern and southern range edges^67^. Maximum size and age is approximately 700 mm total length (L_T_)^66^ and 25 years^41^. It is protogynous, with females at the centre of its range typically attaining sexual maturity at ~3 years^60^, and later changing to males when they are 12 years^59^. Reproduction peaks during the austral spring to mid-summer (October to January) and larvae typically settle onto the reef in the austral summer and early autumn (December to March)^18, 59^. Juveniles and adults are essentially sedentary^68^, but connectivity between populations is maintained via dispersive planktonic larvae with a pelagic larval duration (PLD) of ~23 days (Supplementary Fig. S1).

### Sample collection and DNA extraction

We sampled *C*. *rubescens* at four locations using spears or hook-and-line from October 2012 to April 2013 (Table 1), in accordance with guidelines approved by the University of Western Australia Animal Ethics Committee (Approval no. RA/3/100/1180). Sampling was conducted from two thermally distinct areas of the species’ distribution (3 °C temperature range) separated by approximately 500 km and including geographically distinct populations along the mainland and the offshore Abrolhos Islands (~60 km from the mainland), where the species’ abundance is highest^67^ (Fig. 1). Adult collections (350 to 634 mm L_T_) were undertaken along a 2 km sampling area at seven reefs (2 to 35 m water depth) in three locations: Kalbarri, Abrolhos Islands and Cape Naturaliste (Table 1, Fig. 1). Recruits (L_T_ ≤ 130 mm; 0 + yrs., see Supplementary Fig. S1 for age estimation) were sampled at Garden Island following an unusually high recruitment event at this location towards the cooler (southern) edge of its range^18^. Despite extensive efforts, no adult *C*. *rubescens* were found at this location, possibly because of a combination of low abundance at the southern (cooler) range edge and high fishing pressure. Sampling recruits and adults from the same location is ideal and highly desired, however this may be challenging for many species due to the above reasons and also because recruits often occupy different habitats to adults. Tissue samples were obtained from fin clips and/or muscle tissue of each individual, preserved in 100% ethanol and stored at room temperature. Total genomic DNA was isolated for each sample using DNeasy blood and tissue kits (Qiagen, Valencia, CA, USA) following the manufacturer’s protocols.

### Marker genotyping

Single-nucleotide polymorphism (SNP) loci were genotyped at the ACRF Biomolecular Resource Facility, John Curtin School of Medical Research (JCSMR), Australian National University (ANU) via the genotyping by sequencing (GBS) method^69, 70^. The GBS method is a procedure based on high-throughput next-generation sequencing of genomic subsets targeted by restriction enzymes, and is ideal for non-model organisms that lack genomic resources^70^. Libraries were sequenced with paired-end 100-bp reads on an Illumina HiSeq. 2000 sequencer (Illumina Inc., San Diego, CA, USA). SNP calling was then performed using the TASSEL UNEAK pipeline with default settings^71, 72^. We then selected only loci with a genotyping success rate greater than 85% and retained those for further analyses.

### Detecting outlier loci

To identify loci under directional selection, we used the *FDIST* approach of Beaumont and Nichols (1996)^73^ as implemented in LOSITAN^31^. This method uses expected heterozygosities and unbiased *F*_ST_ values for each locus^74^ to generate a global neutral distribution for *F*_ST_ under Wright’s Island model^75^. The probability of each locus *F*_ST_ belonging to this neutral distribution is then used to classify loci into one of three selection categories: neutral (0.1–0.9), under balancing selection (<0.1) and under positive selection (>0.995)^25^. If a large dataset of neutral loci is used to create empirical *p*-values, this method has high performance and considerably reduces the amount of false-positives^76, 77^. Outlier analyses were based on 1 000 000 simulations assuming an infinite alleles mutation model and using Neutral mean *F*_ST_, 0.95 confidence intervals and an FDR of 0.1. Ten independent runs were performed to further reduce the amount of false-positives. Outlier analyses were performed on adult fish only (Kalbarri, Abrolhos Islands and Cape Naturaliste). Two separate datasets for all samples (adults and recruits) were then generated: one dataset for neutral loci and another for outlier loci under positive selection.

We also identified outlier loci in adult fish by applying a more rigorous Bayesian simulation-based approach implemented in BAYESCAN 2.1 with default settings and an FDR of 0.1. This approach identifies outlier loci under possible natural selection by using differences in allele frequencies between populations and directly estimating the probability that each locus is subject to selection^33^. Furthermore, we used BAYESCENV^34^, a genome-scan method that extends the BAYESCAN approach by including environmental data and using it to identify outliers, based on specific hypothesis about the drivers of local adaptation. We used satellite derived monthly average sea surface temperature data at the sampling unit level (reef) for 2002–2013 (MODIS-AQUA satellite, Goddard Earth Sciences Data and Information Services Center GES DISC, NASA) to carry out a genome scan and identify outliers significantly correlated with the temperature gradient sampled. We used default settings in BAYESCENV, tested for model convergence and set the FDR to 0.1. Temperature has been identified as the most important variable determining fish distributions along the coast of WA, where physiological gradients are consistent and the seascape is climatically buffered, relatively stable in geological time-scales and highly oligotrophic^53^. For this reason and given the relatively limited scope of our sampling and low sample sizes along the WA coast, we chose to limit our linkage of genotypic differences at outlier loci to temperature, rather than undertaking a more detailed landscape approach^54^.

### Population genetic structure

We tested for significant deviations from Hardy-Weinberg equilibrium (HWE) for each neutral locus using the ‘adegenet’ package^78^ in R^79^ and 100 simulations following Monte-Carlo permutation procedures. If loci deviated significantly (*p* ≤ 0.05) from HWE in all regions, they were removed from the SNP data set. We also estimated the extent of linkage disequilibrium (LD) between pairs of loci by calculating significance values for each pairwise comparison and assessing the correlation coefficient for each comparison, using the ‘genetics’ package^80^ in R^79^. In order to correct *p*-values for multiple comparisons and control false discovery rates, we used the *p*.*adjust* function selecting the Benjamini & Hochberg correction method^32^.

To investigate population structure, individual-based discriminant analysis of principal components (DAPC) was conducted for neutral and outlier SNP data as identified via LOSITAN in R, using the package ‘adegenet’^35, 81^. The DAPC method identifies and describes clusters of genetically related individuals from large datasets and allows the optimal visualization of between-population differentiation in multivariate space^82^. Furthermore, by using Bayesian Information Criterion (BIC) to assess the best supported model for identifying groups of individuals, the method provides a measure of the optimal number of genetic clusters (*K*) across a range of *K* values^82^. We used both the outlier and neutral loci datasets to test for population structure, and selected the model with the lowest BIC to identify the optimal number of *K*. The optimal number of principle components and discriminant functions to use in DAPC plots was determined by maximization of the *α*-*score*, which measures the bias between observed and random discrimination and provides a measure of discrimination ability and stability of the DAPC^35^. For all analyses we retained five principal components (PCA) and five linear discriminants (DA).

In addition to the DAPC analyses, Bayesian inference of genetic partitioning was implemented in the program STRUCTURE^39^ with a burn-in period of 10 000 and 100 000 MCMC iterations, and under the assumption that populations were admixed and allele frequencies correlated between populations. Analyses were run for *K* = 1 to 5, each replicated 10 independent times and without assuming prior genetic structure. The most likely number of genetic clusters (*K*) was chosen based on results from STRUCTURE HARVESTER^37^ by comparing the likelihood of the data for different values of *K* and using the *ΔK* method^83^. Results were then averaged using CLUMPP to minimize variance across iterations^84^, before graphics were generated.

### Assignment of recruits

Recruits were assigned to parent populations by assessing membership probabilities of individual juveniles (14 individuals) to different groups and the proximity of individuals to the different clusters (*K*) identified via BIC in both DAPC analyses^82^ and STRUCTURE^37^, as described in the previous section. Outlier loci identified via LOSITAN were used for this assignment. For DAPC analyses, ordination plots were produced with and without a-priori information of different values of *K* in order to better visualize recruit assignment.

### Gene function of outlier loci

To determine the possible function of outlier loci, we examined loci identified via any of the three outlier detection methods used (LOSITAN, BAYESCAN and BAYESCENV; all assuming an FDR of 0.1) for alignment with publicly available genomes using the NCBI program BLASTn. We followed Gaither *et al*. guidelines^85^ that considered an alignment significant if a sequence match of ≥50% and an E-value of ≤e-10 was found. We then used GenBank’s non-redundant protein database (NR) and Uniprot’s Swiss-Prot databases to query BLASTn top hits. The number of significant matches did not change greatly when a less conservative E-value was used (≤e-5, three extra matches).

## Electronic supplementary material

Supplementary Information
