# Supplementary material for: Genomic signatures of local adaptation reveal source-sink dynamics in a high gene flow fish species
Source: Sci Rep. 2017 Aug 17;7:8618. doi: 10.1038/s41598-017-09224-y (PMC5561064; doi:10.1038/s41598-017-09224-y)
Supplement: Supplementary file 1 — Supplementary Information [file 41598_2017_9224_MOESM1_ESM.pdf]

Supplementary Information

Genomic signatures of local adaptation reveal source-sink dynamics in a high gene flow fish species

Katherine Cure[1,2*], Luke Thomas[1,3], Jean-Paul A. Hobbs[4], David V. Fairclough[5], and W. Jason Kennington[6]

[1] UWA Oceans Institute & School of Plant Biology, The University of Western Australia, Crawley, 6009, Australia , [2] Australian Institute of Marine Science, Crawley, 6009, Australia , [3] Hopkins Marine Station, Stanford University, California, 93950, USA , [4]Department of Environment and Agriculture, Curtin University, Bentley, 6102, Australia , [5] Western Australian Fisheries and Marine Research Laboratories, Department of Fisheries, Hillarys, 6025, Australia , [6] Centre for Evolutionary Biology, School of Animal Biology, The University of Western Australia, Crawley, 6009, Australia

Supplementary Methods: age and pelagic larval duration estimation

Juvenile *Choerodon rubescens* (n =14) were collected using hand-spear while free-diving in shallow water lagoons (<3 m) at Garden Island, Western Australia (32.1184 °S, 115.6627 °E) during January 2013. Each captured fish was measured to the nearest mm total length (L_T), and sagittal otoliths removed, cleaned in ethanol and stored dry. Otolith processing was performed as described in Choat et al. (2003), with all sections ground and polished by hand with lapping film (0.3-3 μ). Individual ages (days) were estimated by counting daily opaque zones on transverse sections of sagittal otoliths under a compound microscope and 40× magnification. Blind reads of daily increments were performed twice by a single observer, and validated by an experienced otolith reader. Each read was done with at least a week in between. If the error between the first two reads was more than 10%, a third blind read was conducted. If the closest of the three reads differed by more than 10%, the sample was excluded from analyses. When counts were accepted, an average of the two closest counts was used as the estimate for age. The number of daily increments from the nucleus to the settlement mark was counted to estimate pelagic larval duration (PLD) following Takahashi et al. (2012). PLD was estimated in a subset of specimens with high clarity of nucleus and definition of settlement mark. Daily estimates of age and PLD were then used to back-calculate settlement and birth date for all possible specimens.

References

Choat, J., Robertson, D., Ackerman, J. & Posada, J. An age-based demographic analysis of the Caribbean stoplight parrotfish *Sparisoma viride*. *Mar. Ecol. Prog. Ser.* **246**, 265–277 (2003).

Takahashi, M., McCormick, M. I., Munday, P. L. & Jones, G. P. Influence of seasonal and latitudinal temperature variation on early life-history traits of a coral reef fish. *Mar. Freshw. Res.* **63**, 856–864 (2012).

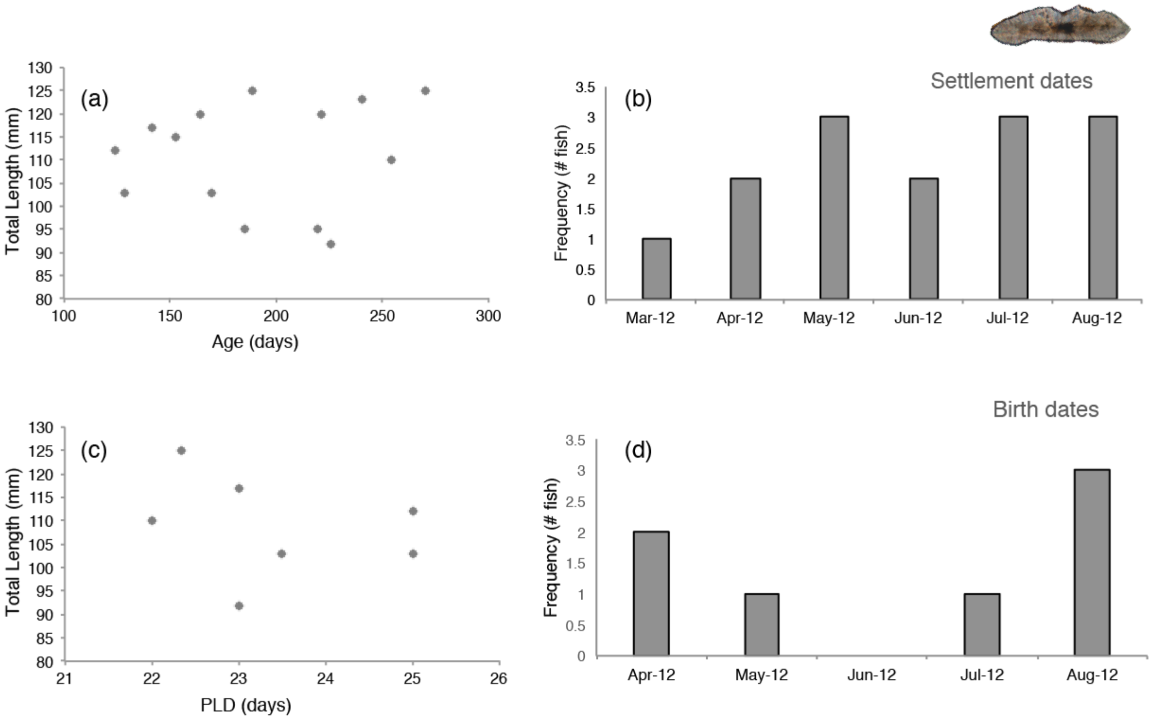

Supplementary Figure S1. Ageing and back-calculation of settlement and birth dates of *Choerodon rubescens* recruits: (a) post-settlement age (days) *vs*. fish size (total length in mm, L_T), (b) frequency distribution of settlement dates for aged fish, (c) pelagic larval duration (PLD) for recruits where PLD estimation was possible (n = 7) and (d) frequency distribution of birth dates from total age (post-settlement age + PLD) of recruits where PLD estimation was possible. Image above (b) is a transverse section of a sagittal otolith.

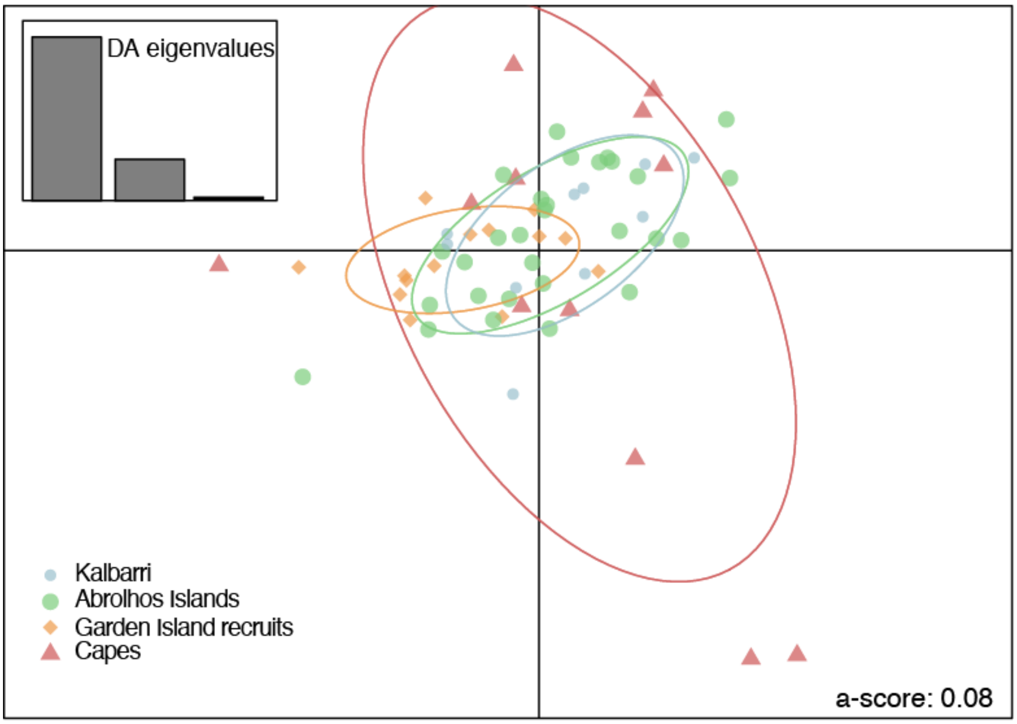

Supplementary Figure S2. Discriminant analysis of principle components (DAPC) with associated alpha score (a-score) showing the structure of neutral loci (n = 11 055) from *Choerodon rubescens* populations; eigenvalues show the relative amount of genetic information contained in each successive principal component; $K = 1$ from Bayesian analyses (BIC), see Figure S3 for K selection information. Kalbarri, Abrolhos Islands, and Cape Naturaliste populations are composed of adult fish (≥350 mm L_T), whereas the population at Garden Island is represented exclusively by recruits (≤130 mm L_T, 0+yrs).

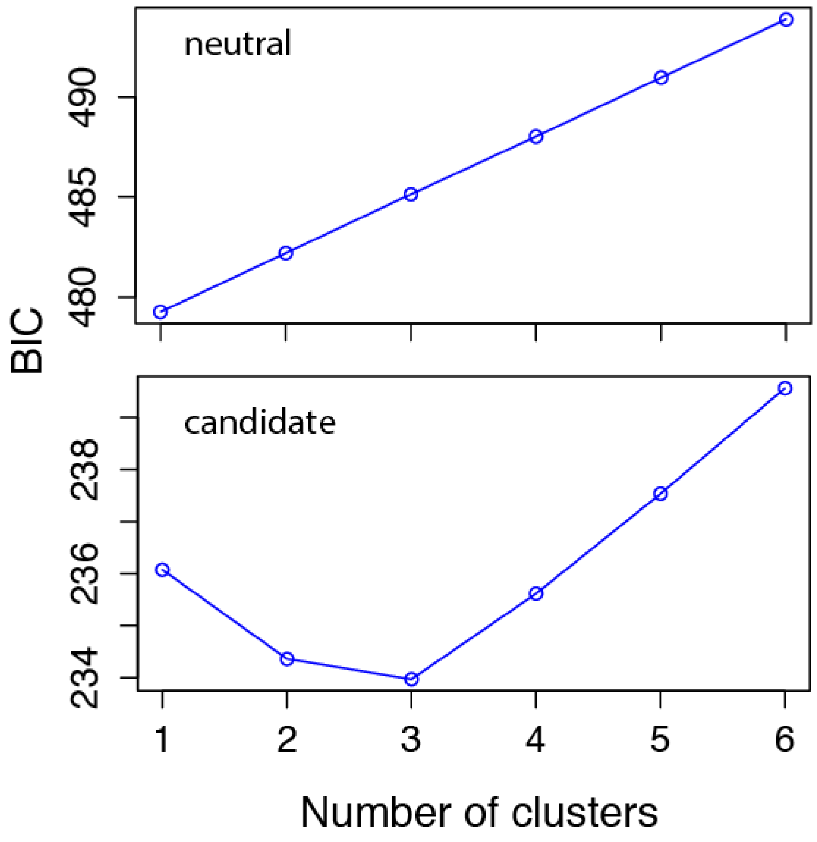

Supplementary Figure S3. Results from the Bayesian information criterion (BIC) method used to identify the optimal number of genetic clusters in neutral ($K = 1$; above) and outlier loci ($K = 3$; below) of the SNP dataset for *Choerodon rubescens*. Plots show BIC values across different levels of K (Number of clusters).

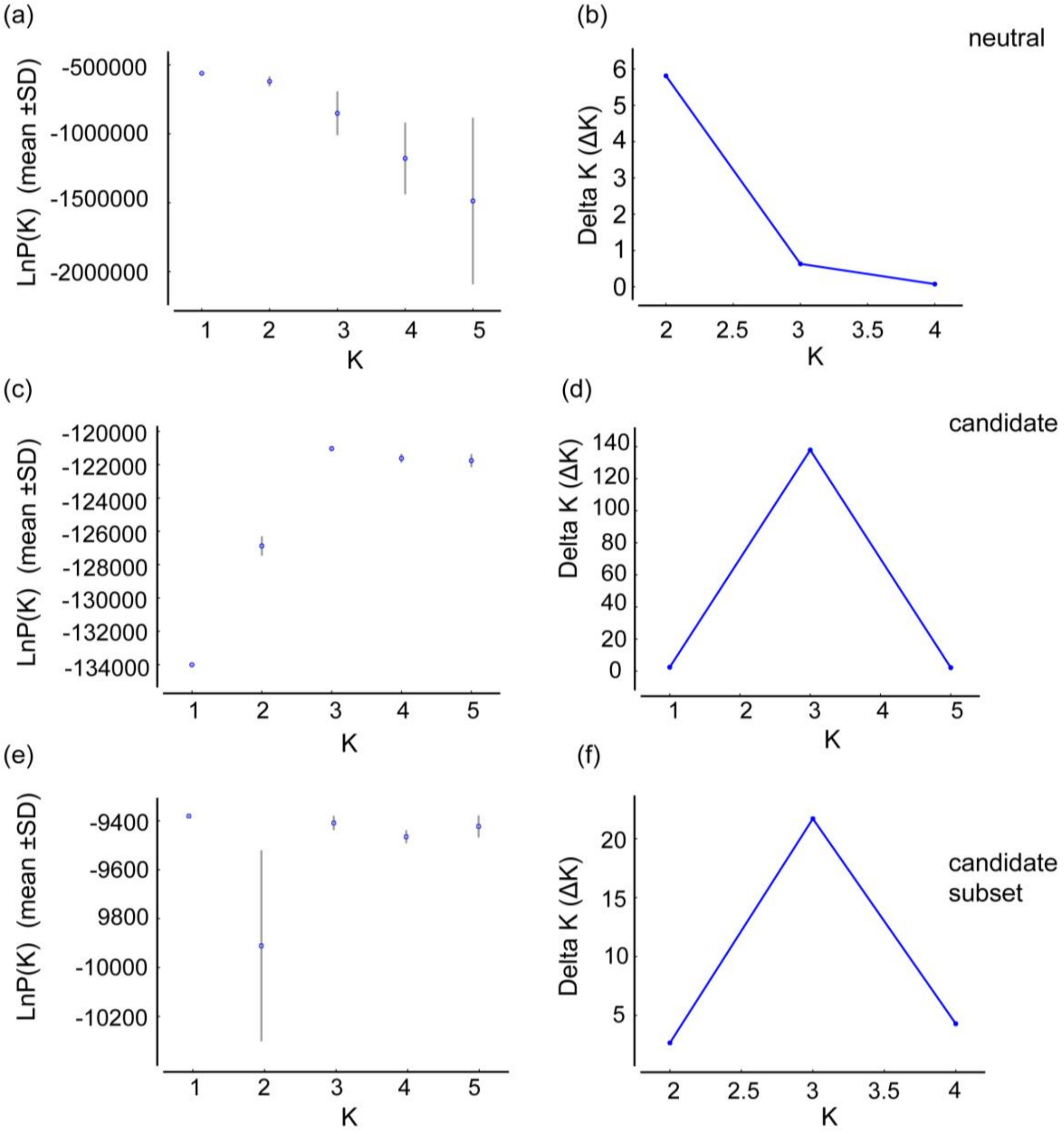

Supplementary Figure S4. Results from Evanno method for selection of optimal number of clusters from STRUCTURE analyses with *K* ranging from 0 to 5 for neutral loci (a,b), outlier loci (c,d), and outlier loci from a subset of samples (Garden Island recruits and Abrolhos Island adults, e,f): (a), (c) and (e) show log probability of *K* (LnP(*K*)) across different values of *K*; (b), (d) and (f) illustrate delta *K* (*ΔK*) across different values of *K*. This method resulted in the selection of *K* = 1 for neutral loci, *K* = 3 for outlier loci (see Figure S2), and *K* = 1 for the subset of outlier loci from Garden Island recruit and Abrolhos Island adult populations.

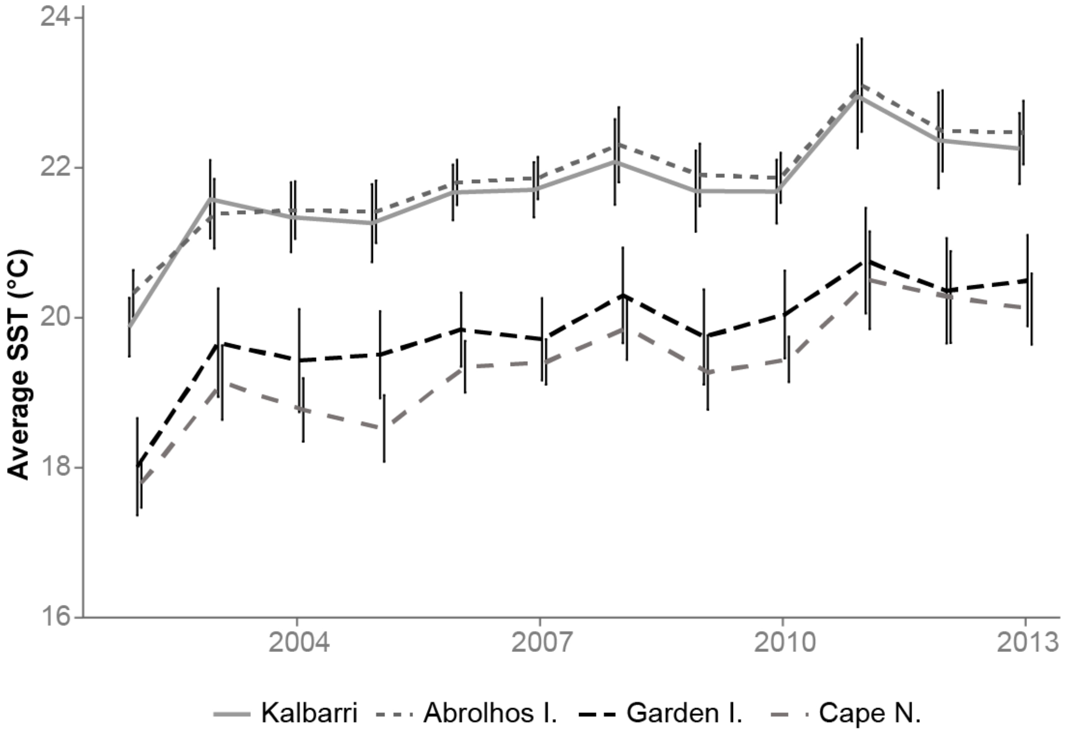

Supplementary Figure S5. Time series of average monthly sea surface temperatures (SST °C) at locations sampled along the geographical range of *Choerodon rubescens* in Western Australia. Shown are annual means ±SE from 2002 to 2013 at each of the four locations sampled (Kalbarri, Abrolhos Islands, Garden Island and Cape Naturaliste). Data are satellite-derived temperatures from MODIS-AQUA at a 4 km resolution (Goddard Earth Sciences Data and Information Services Center GES DISC, NASA).
